# Challenges of total hip replacement surgery in austere settings: a case series of 158 patients operated in the Far North Region of Cameroon

**DOI:** 10.11604/pamj.2025.50.66.46467

**Published:** 2025-03-06

**Authors:** Berenger Tsanga Bessala, Cédric Dongmo Mayopa, Lionel Kolontchang, Gaspary Fodjeu, Chifen Umaru, Randy Buzisa Mbuku, Franck Biongolo, Daniel Handy Eone, Jean Bahebeck

**Affiliations:** 1 Department of Surgery and Specialty, Faculty of Medicine and Biomedical Sciences, University of Yaounde I, Melen, Yaounde, Cameroon; 2 Orthopedic and Trauma Unit, Maroua Military Hospital, Maroua, Cameroon,; 3 Department of Orthopaedic Surgery and Traumatology, *Cliniques Universitaires* Saint-Luc, Avenue Hippocrate, Brussels, Belgium; 4 UCLouvain - IREC, Neuromusculoskeletal Laboratory (NMSK), Avenue Emmanuel Mounier, Brussels, Belgium; 5 Orthopedic and Trauma Unit, Yaoundé Central Hospital, Yaoundé, Cameroon; 6 Surgery Unit, District Hospital of Efoulan, Yaoundé, Cameroon

**Keywords:** Total hip arthroplasty, resource-limited settings, traditional medicine, postoperative complications, humanitarian surgery

## Abstract

The incidence of total hip replacement (THR) surgery is on the rise in Cameroon, attributed to the growing number of practitioners, increased availability of implants, and continuous improvement in surgeons' skills. However, challenges persist, particularly in the operating environment, equipment availability, financial policies, and prevailing cultural beliefs regarding traditional medicine. This study offers a short-term review of primary hip arthroplasty activities in an austere setting of the Far North region of Cameroon. A total of 168 hip arthroplasties were performed on 158 patients at the Maroua Military Hospital over 72 months. All procedures were done by a single surgeon using the anterior Hueter approach. Data on socio-demographics, clinical and radiological findings, and intraoperative details were collected. Complications and functional outcomes were assessed at a mean follow-up of 16 months using the Postel-Merle d'Aubigné (PMA)score. The study included 111 men and 47 women, with an average age of 49.39 years. Most patients (85.45%) had low to moderate incomes, and 79.75% of surgeries were financed in two or three installments. The main indications for surgery were neglected trauma and end-stage avascular necrosis, often delayed by traditional medical practices. Intraoperative complications, mainly nerve damage, occurred in 8% of cases, while postoperative infections were seen in 8.8%. At the final evaluation, 65% of patients had good or excellent outcomes according to the PMA score. This study highlights the challenges of performing total hip replacement surgery in resource-limited settings. It suggests improving technical infrastructure and introducing progressive funding options to enhance outcomes and reduce complication rates.

## Introduction

Total hip arthroplasty (THA) is the conventional treatment for end-stage hip diseases. This surgical procedure aims to restore a mobile, pain-free hip and has achieved significant success over the years [1]. In Cameroon, hip replacement surgery is expanding significantly, with the number of practitioners rising from approximately thirty in 2013 to around a hundred in 2023.

Over the past decade, improvements have been made concerning the availability of implants, surgical instrumentation, and surgeons' skills, including adopting minimally invasive approaches, potentially leading to better early functional outcomes [2,3]. However, several challenges persist, particularly regarding the operating environment, which necessitates fully equipped operating theatres dedicated exclusively to orthopaedic surgical procedures requiring exceptionally high aseptic conditions [4,5]. There is also a need to improve financial policies to make hip replacement procedures more affordable for low-income populations and to change cultural beliefs about traditional medicine, which delays consultation and complicates management [6].

This study presents a short-term review of primary hip arthroplasty activities in an austere setting in the Far North Region of Cameroon, highlighting the challenges surrounding the practice of this surgery in limited settings.

## Methods

**Study design:** a cross-sectional study was conducted to describe the current challenges of THR surgeries in the Far North Region of Cameroon.

**Study setting and population:** the Far North Region of Cameroon is one of the ten administrative regions of the nation. It shares borders with the countries of Chad and Nigeria. The region is subdivided into six departments and covers an area of 34,246 square kilometers. It is home to more than 2,721,500 inhabitants, which makes it the densest region of the country.

**Figure 1:**
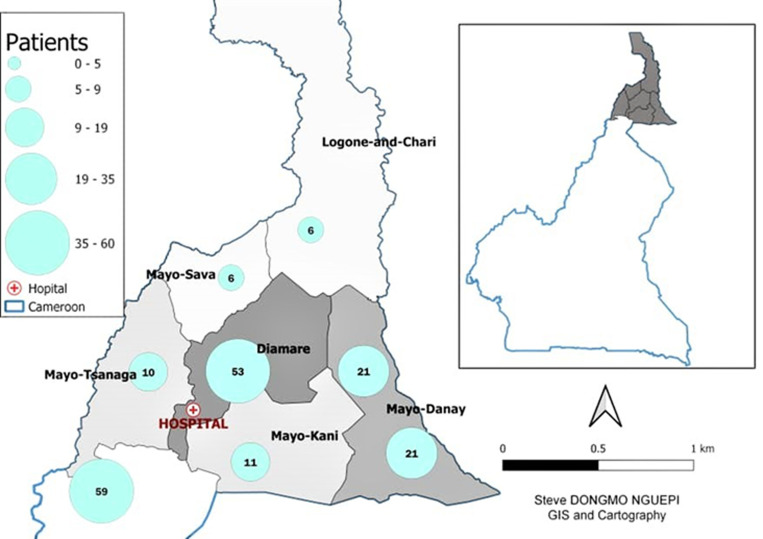
population mapping of hip arthroplasty patients in the Far North Region of Cameroon (N=187), 2024

The study population was Far North Region habitants aged above 16 years old suffering from hip diseases. The study started with information campaigns on the benefits of modern orthopedic treatment for hip diseases across six departments in the Far North Region of Cameroon (Figure 1) from January 2020 to June 2023. A sequential funding model, based on trust and commitment under the guidance of local authorities (Lamido, Imam, and Priest) was proposed to patients suffering from hip disease with indication of total hip replacement. 187 patients underwent hip surgery at the Maroua Military Hospital, including 158 patients for primary total hip arthroplasty (including 10 cases of bilateral cases for a total of 168 total hip arthroplasties) and 29 cases of arthroplasty complications from surgeries performed at other hospitals.

The same surgeon performed all surgeries. The main approach for hip arthroplasty was the anterior Hueter approach using the two-limb preparation technique without a traction table. A minority of patients underwent different approaches (lateral or posterior), depending on preoperative clinical and radiologic presentations. Uncemented femoral stems and acetabular metal back were used in all patients. A polyethylene-metal friction torque was used in all cases. A postoperative follow-up was conducted with a mean follow-up duration of 16.5 months (range: 1 to 43 months).

**Variables:** they included socio-demographic information, pre-and postoperative radiological and clinical data, and intraoperative information such as operation duration, amount of bleeding, complications, and functional outcomes.

### Data resource and measurement

**Data collection tool:** we designed and used a data collection form that included socio-demographic and clinical characteristics (pre- and post-operatively), as well as the *Postel Merle d'Aubigné* Functional Assessment Score questionnaire [7].

**Data collection:** socio-demographic data included age, sex, financial income, and mode of financing surgery. Clinical data included indication for surgery, side affected, surgical approach, type of implants used, bleeding, duration of surgery, and intraoperative incidents, as well as postoperative data such as immediate and early complications (infection, fractures, deep vein thrombosis) and functional score.

**Sample size:** all patients over 18 years of age with degenerative hip disease in the 6 departments of the Far North Region of Cameroon were included in this study (Figure 1), with a final sample size of 187 patients, including 158 patients for primary total hip arthroplasty (10 cases of bilateral arthroplasty for a total of 168 total hip arthroplasties) and 29 cases of arthroplasty complications from surgeries performed at other hospitals.

**Data analysis:** statistical analyses were performed using IBM SPSS 27.0.1.0. Qualitative data were presented in numbers and percentages, and quantitative data in means, medians, and standard deviations.

**Ethical consideration:** this study was approved by the University of Yaounde 1 Research Ethical Committee (No: 321, December 2019). All patients over 18 years of age were asked to give informed consent and sign a consent form before participating in the study. No personally identifiable information was collected, and confidentiality was ensured. The electronic data has been stored in a password-protected computer.

## Results

**Socio-demographic characteristics:** among the 158 patients, 111 (70.25%) were men, and 47 (29.75%) were women, with a sex ratio of 2.4. The mean age was 49.39 years (SD: 16.24). A significant proportion, 135 (85.45%), had low or average income, with 126 (79.75%) procedures financed in two or three payment stages (Table 1).

**Table 1:** socio-demographic characteristics of patients undergoing total hip replacement in the Far North of Cameroon

Socio-demographic features	Effective (n=158)	Percentage (%)
**Sex**		
Men	111	70.25
Women	47	29.75
Sex-ratio	2.4	
**Age**		
Mean	49.39	
Minimum	16	
Maximum	103	
SD*	16.24	
**Socio-economic classes**		
Low	81	51.27
Middle	54	34.18
High	23	14.56
**Mode of financing**		
One-stage financing	32	20.25
Two-stage financing	58	36.71
Three-stage financing	68	43.04

* SD: standard deviation

**Figure 2:**
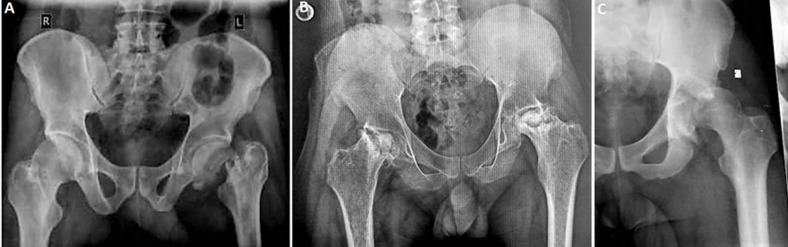
common indications for total hip replacement: A) neglected left femoral neck fracture; B) bilateral femoral head necrosis in terminal stage; C) neglected posterior dislocation of the left hip, Maroua Military Hospital, Cameroon, 2024

**Clinical and intraoperative characteristics:** the primary indications for hip replacement were osteoarthritis or avascular necrosis of the femoral head (AVN) in 83 (52.54%) patients and neglected trauma (Figure 2), mainly hip fractures or dislocations, in 55 (34.81%) patients (Table 2). Additionally, 18 cases of neglected paediatric hip diseases (Perthes disease, slipped femoral epiphysis, and developmental dysplasia of the hip) and two cases of hip tuberculosis were also observed.

**Table 2:** clinical and intraoperative characteristics, complications, and functional results

Primary total hip arthroplasty	Effective (n)	Percentages (%)
**Indications**	**n=158**	
Hip osteoarthritis	26	16.46
AVN*	57	36.08
Trauma**	55	34.81
Pediatric hip diseases***	18	11.39
Hip tuberculosis	2	1.27
**Operated side**		
Left	63	39.87
Right	85	53.80
Bilateral	10	6.33
**Approach**	**n=168**	
Anterior (Hueter)	150	89.29
Latera l(Hardinge)	13	7.74
Posterior (Moore)	5	2.98
**Duration**		**(Minutes)**
Mean	77.1	
Min	52	
Max	135	
Intraoperative complications	38	22.62
Fractures	8	4.76
Fat embolisms	5	2.98
Nerve injury	13	7.74
Bleeding	6	3.57
Acetabulum cup malposition	1	0.60
Femoral stem malposition	5	2.98
Postoperative complications	25	14.88
Infection	14	8.33
Dislocation	2	1.19
Fracture	2	1.19
Loosening	2	1.19
Deep vein thrombosis	5	2.98
**Preoperative mean: 7.7 (min=3; max=13)**		
**PMA score (n=158)**		
**Postoperative mean: 16.8 (min=7; max=18)**		
Excellent (18)	22	13.92
Good (15-7)	84	53.16
Fair (12-14)	38	24.05
Poor (<12)	14	8.86

AVN*: avascular necrosis; trauma**: cervical neck fracture and hip dislocation; pediatric hip diseases***: Perthes disease, slipped femoral epiphysis, and developmental dysplasia of the hip; PMA: *Postel Merle D’Aubigné score*

**Figure 3:**
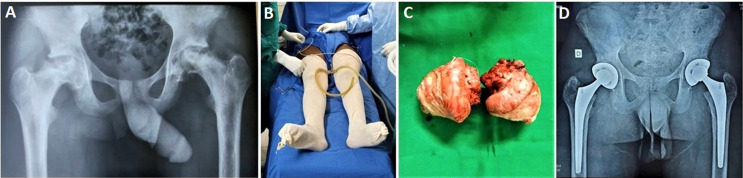
bilateral total hip arthroplasty indicated for end-stage osteoarthritis: A) preoperative X-ray; B) installation on the operating table (double draping); C) enucleation of the two femoral heads; D) postoperative X-ray, Maroua Military Hospital, Cameroon, 2024

**Figure 4:**
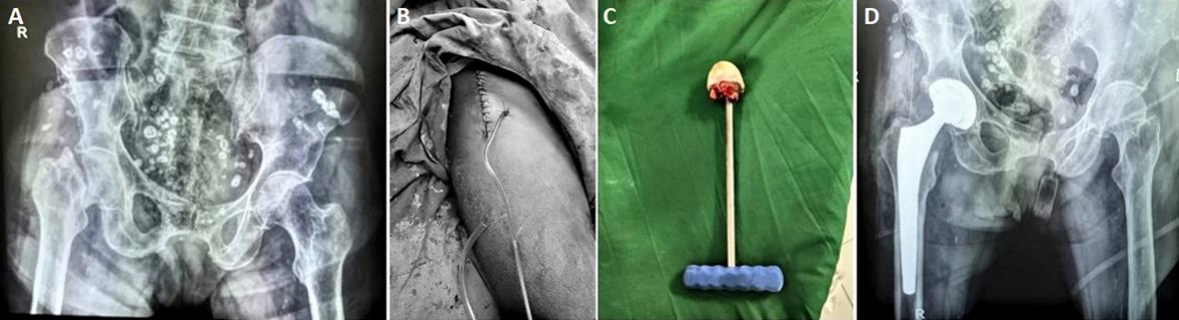
total hip arthroplasty for a neglected femoral neck fracture: A) preoperative X-ray; B) surgical wound and tubular drain; C) femoral head; D) postoperative X-ray, Maroua Military Hospital, Cameroon, 2024

Of this cohort of patients, 85 (53.80%) underwent surgery on the right side, 63 (39.87%) on the left, and 10 (6.33%) had bilateral hip replacement, with four bilateral cases operated on in a single session (Figure 3). The direct anterior approach (Hueter's) was employed in 150 patients (89.29%), while posterior and lateral approaches were used in 18 patients (10.72%). A closed-circuit vacuum drain was employed for the systematic drainage of surgical wounds (Figure 4). The mean operating time was 77 minutes, with a range from 52 to 135 minutes.

**Figure 5:**
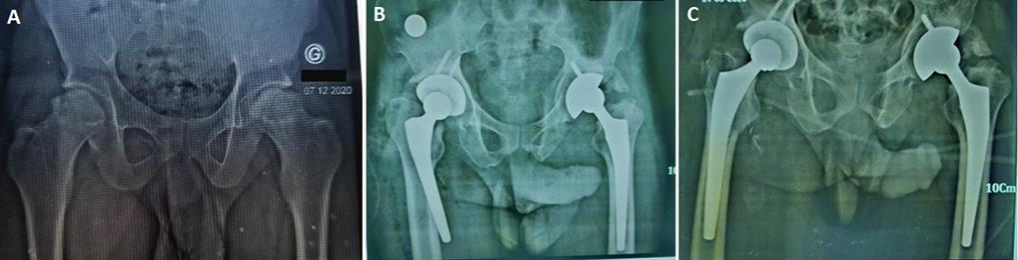
intraoperative complications: A) preoperative X-ray; B) femoral stem malpositioning on the right; C) surgical revision to reposition the femoral stem, Maroua Military Hospital, Cameroon, 2024

Intraoperative complications were observed in 38 cases (22.62%), with the majority of these being femorocutaneous nerve injuries (13 cases, 7.74%), followed by proximal or femoral shaft fractures (8 cases, 4.76%) and severe bleeding necessitating blood transfusion (6 cases, 3.57%). Additionally, five cases (2.98%) manifested symptoms of fat embolism. Implant malposition was observed in six instances, comprising five instances of femoral stem malposition (Figure 5) and one instance of acetabular malposition.

**Postoperative characteristics:** the mean postoperative follow-up period was 16.5 months, during which 25 patients (14.88%) experienced postoperative complications (Table 2). These included 14 cases of surgical site infections (8.33%), five reports of deep vein thrombosis (2.98%), and six mechanical complications (3.57%), including dislocation, fractures, and loosening. At the final functional evaluation, 106 patients (67.09%) exhibited an excellent or good PMA score, while 14 patients (8.86%) demonstrated a poor PMA score.

## Discussion

The Far North Region of Cameroon is one of the most vulnerable and insecure areas in the country, with limited access to basic services such as water, education, and healthcare. Extreme climatic conditions, alternating between drought and flooding, destroy crops and foster food insecurity, exacerbated by an unstable security situation due to terrorism since 2014 [8,9].

Due to these difficulties, particularly the lack of access to quality care, people often resort to traditional practitioners. These practitioners are geographically and culturally closer to the population, operating on a sequential, staggered financing model based on trust. This financing method is better suited to the population's variable incomes, unlike the state health system, which requires full payment before providing care in the absence of private insurance [10].

By implementing public education programs on the management of hip pathologies at medical facilities and replicating the staggered financing model, we were able to treat 187 patients with hip pathologies over approximately three years. The majority of patients financed their surgery in two or three sequences. This frequency exceeds that observed in the country's reference hospitals. Fonkoue *et al*. [11] recorded 130 cases of total hip arthroplasty over a five-year period in five different medical institutions in Yaounde City.

The management of musculoskeletal conditions by traditional practitioners often results in a delay in appropriate treatment, leading to complications or severe forms [6,12]. Thus, our series included mainly end-stage forms of degenerative hip disease and major neglected hip trauma (fracture or dislocation) after unsuccessful attempts by traditional practitioners.

We recorded a rate of 22.62% of intraoperative complications, some of which were directly related to the surgical technique. These included damage to the lateral femoral cutaneous nerve (LCFN), clinically manifested as numbness and/or dysesthesia of the lateral aspect of the thigh (meralgia paresthetica), fractures of the proximal femur and diaphysis, and a few cases of severe bleeding requiring blood transfusion and implant malposition. The rate of major intraoperative complications in high-level care centers can be less than 1% [13]. The discrepancy in complication rates can be attributed to two main factors: the limited experience of our current surgical team, who are still developing their expertise without the input of specialized surgical nurses or instrumentalists; and the lack of adequate technical resources in our operating theatres. Specifically, the lack of an image intensifier prevents intraoperative verification of implant positioning, potentially contributing to complications (Figure 5). There is a wide variation in the incidence of LCFN injury in hip replacement surgery, ranging from 0.1% to 81% [14]. This may be explained by the proximity and crossing of the nerve in the interval between the Sartorius and tensor fascia lata muscles, which is injured when this space is opened during the direct anterior approach [14]. Intraoperative femoral fractures occur with an incidence of between 0.6% and 6% under optimal conditions [15] and usually occur during the preparation of the femoral canal, regardless of the approach.

Our postoperative complications were dominated by a high incidence of surgical site infections, but similar to those found in other local series (8.6% for Fonkoue *et al*.) [11]. These values are almost four to eight times higher than current standards (1-2%) [16], likely due to the surgical environment not meeting current standards [16]. Our operating theatre is not exclusively dedicated to clean surgeries; maintenance of the sterilization chain is questionable, air circulation flow devices are lacking, and room temperature regulation is suboptimal [17]. Devices such as Ioban-type adhesive incision fields, designed to optimize sterility conditions [18], are also unavailable.

Our mechanical complication rates are comparable to other studies in institutions with adequate technical facilities, where mechanical complication rates for the direct anterior approach vary between 0.96% and 1.5% for dislocation [19] and 3.4% for aseptic loosening [20]. The functional results were excellent or good in almost 67% of patients. This encouraging outcome was achieved through a series of adaptations designed to circumvent the inherent limitations of the local context. It reflects the complexity of neglected cases but, at the same time, the dedication of the surgical team to address hip pathologies affecting underprivileged populations in this region of Cameroon. However, it also highlights the necessity for further improvements.

It is important to acknowledge several limitations of this study. Firstly, the relatively short postoperative follow-up period makes it impossible to study late complications. However, this does not have a significant impact on the objective of our study, which is to address the practical challenges of performing prosthetic hip replacement surgery in our context. in addition, the design of this study only provides an overview of the challenges, without allowing for any in-depth understanding due to the absence of an analytical component.

## Conclusion

This study highlights the challenges of humanitarian surgery specific to THR surgery in austere settings. It emphasizes the barriers to accessing quality care due to the unpredictability of the population's financial income. The findings of this study indicate that access to this expensive surgical procedure can be enhanced through a strategy of sequenced and progressive funding. Despite the elevated complication rate in comparison to current standards, it is possible to mitigate this through the strengthening of technical infrastructure. The sustained investment in surgical equipment and financial policies, in addition to the addressing of cultural beliefs, are crucial elements in the improvement of success rates for this procedure.

### What is known about this topic

- Total hip replacements are increasingly being performed in Cameroon;
- Access to THR is significantly restricted for low-income populations;
- Under optimal conditions, THR is an effective procedure with a low complication rate.

### What this study adds

- Access to THR for disadvantaged populations can be facilitated by sequential financing;
- Neglected traumatic hip pathologies and end-stage avascular necrosis represent the predominant indications for THR in austere environments;
- This series is the largest of all the studies carried out on total hip prostheses in Cameroon.
